# Oral vancomycin desensitisation to treat Clostridium difficile infection in a vancomycin allergic patient

**DOI:** 10.1186/1939-4551-6-16

**Published:** 2013-09-30

**Authors:** Shanti Mahabir, Ren Yik Lim, Fidelma Fitzpatrick, Colm Magee, Mary Keogan

**Affiliations:** 1 Immunology Department, Beaumont Hospital, Dublin 9, Ireland; 2 Department of Renal Medicine, Beaumont Hospital, Dublin 9, Ireland; 3 Microbiology Department, Beaumont Hospital, Dublin, Ireland; 4 Health Protection Surveillance Centre, Dublin, Ireland

**Keywords:** Allergy, Anaphylaxis, Desensitisation, Vancomycin, Clostridium difficile

## Abstract

The prevalence of Clostridium difficile infection (CDI) is increasing worldwide. Oral vancomycin is an effective and frequently used treatment. However, patients with CDI who are allergic to intravenous vancomycin cannot receive oral vancomycin due to the risk of anaphylaxis if given the oral form.

We present a case where oral vancomycin desensitisation was used to successfully treat a vancomycin allergic patient with recurrent CDI.

## Background

*Clostridium difficile* infection (CDI) is a common healthcare-associated infection associated with a spectrum of disease which includes life threatening complications. CDI recurs after treatment in 15 - 35% of cases [1], defined as an episode of CDI that occurs within 8 weeks following the onset of a previous episode [2].

Vancomycin is recommended rather than metronidazole for the treatment of severe CDI on the basis of higher intracolonic drug concentrations and possibly faster clinical responses [3]. Irish guidelines recommend vancomycin to treat severe CDI and either metronidazole or vancomycin to treat a first recurrence of CDI. The decision to use vancomycin is based on the presence of markers of severe disease [4]. Tapered-pulsed vancomycin is recommended for treatment of multiple (≥ 2 episodes) recurrences of CDI. Administering vancomycin over an extended time period at decreasing doses or via intermittent delivery is thought to work by gradually clearing *C. difficile* by eradicating cells as spores germinate, and may aid in the restoration of normal flora.

Oral vancomycin is poorly absorbed from the gut. However, reports have shown systemic absorption in patients with inflamed colonic mucosa. Serum vancomycin levels causing systemic side effects may be achieved, especially in those with renal impairment. [5,6] Hence, oral vancomycin is contraindicated in those allergic to intravenous vancomycin, due the risk of exposure causing anaphylaxis.

Desensitisation is used when the need for a medication outweighs the risk of reaction during the procedure, and where an alternative is not available or has failed. It involves administering slowly increasing doses and eventually renders mast cells unresponsive to the medication; however, the mechanism of drug desensitisation is not yet well defined. Close monitoring for an allergic reaction is essential, with treatment readily available. Desensitisation is not permanent, as sensitisation may recur once regular exposure to the drug is stopped.

An oral vancomycin desensitisation protocol was developed by modification of a published protocol for intravenous desensitisation [7] and used to successfully treat an elderly patient with recurrent CDI and multiple comorbidities, including end stage renal disease (ESRD), who was allergic to intravenous vancomycin. Desensitisation to intravenous vancomycin has been reported [8,9] but this is the first protocol for oral vancomycin desensitisation to our knowledge.

## Case report

The 65 year old lady had multiple drug allergies, both immediate (ciprofloxacin, cefalexin) and delayed (penicillins, linezolid). She had received vancomycin in 2001 without ill effects. When vancomycin was administered again in 2003, she immediately developed a generalised urticarial rash requiring treatment with antihistamine and hydrocortisone.

She was admitted with a transhepatic line infection and pulmonary embolism. During her admission she developed 6–7 episodes of liquid stool per day with bleeding per rectum. Stool samples were positive for *C.difficile* toxins and the diarrhoea resolved following oral metronidazole treatment for 10 days.

However, diarrhoea and pyrexia recurred 2 weeks later, associated with hypotension, (80/50 mmHg) abdominal tenderness and elevated venous lactate of 2.5 mmol/l (0.9 – 1.7 mmol/l), raising concerns of impending toxic megacolon. Metronidazole was recommenced at 500 mg tid but had no effect, and her C reactive protein remained elevated at 63 showing significant inflammation.

Oral tapered vancomycin therapy was recommended in view of her recurrent CDI with markers of severe infection (hypotension, abdominal tenderness, and elevated serum lactate) and failed metronidazole treatment. It is likely that oral vancomycin treatment would have resulted in systemic absorption sufficient to cause a hypersensitivity reaction as she had both significant bowel inflammation and renal impairment, which contribute to systemic absorption and accumulation of vancomycin. She was therefore offered a trial of oral desensitisation to vancomycin.

Following informed consent she received incremental doses of oral vancomycin at 20 minute intervals (See Table 1). Intravenous access was in situ, emergency medications (epinephrine, hydrocortisone and antihistamine) were drawn up at the bedside and a doctor was present throughout. No premedication was used. The procedure took 5 hours, and there were no adverse events. Following desensitisation, vancomycin was administered orally, initially at a dose of 250 mg qds for 14 days, then tapering to 125 mg qds for 7 days, 125 mg bd for 7 days and 125 mg daily for 7 days. Her symptoms resolved after 2 weeks of treatment and did not recur.

**Table 1:** Oral vancomycin desensitisation protocol

**Fraction of dose**	**Dose given (mg)**	**Cumulative dose (mg)**	**Volume given (ml)**
*Using Solution A, 0.01 mg/ml*			
1/10,000	0.025	0.025	2.5
1/5,000	0.05	0.075	5
1/2,500	0.1	0.175	10
1/1,250	0.2	0.375	20
*Using Solution B, 1 mg/ml*			
1/625	0.4	0.775	0.4
1/312	0.8	1.575	0.8
1/160	1.5	3.075	1.5
1/80	3.0	6.075	3
1/40	6.0	12.075	6
*Using Solution C, 5 mg/ml*			
1/20	12.5	24.575	2.5
1/10	25	49.575	5
1/5	50	99.575	10
1/ 2.5	100	199.575	20
	150	349.575	30
1/ 1.25	200	549.575	40

Make up 1 mg/ml solution (500 mg vancomycin in 500 mls saline or other diluent). This is Solution B.

Make up 0.01 mg/ml solution (Take 1 ml of solution B, dilute it in 100 mls saline.) This is Solution A.

Make up 5 mg/ml solution (500 mg Vancomycin in 100 mls saline or other diluent). This is Solution C.

## Discussion

Our patient was an elderly lady with multiple co-morbidities. She had recurrent CDI with markers of severe infection (hypotension, abdominal tenderness, elevated serum lactate) and had failed metronidazole treatment. She had both renal impairment and significant bowel inflammation, which are risk factors for systemic absorption and accumulation of vancomycin, increasing the possibility of an allergic reaction.

Our patient’s history of previously well tolerated vancomycin administration with immediate development of urticarial rash on subsequent exposure is typical of an IgE mediated allergic reaction. It can be difficult to distinguish between vancomycin – induced anaphylaxis and severe Red Man Syndrome clinically. Anaphylaxis is due to IgE mediated histamine release, whereas RMS is due to release of histamine by a non-IgE mediated mechanism and is dependent on infusion rate. Our hospital policy requires that vancomycin is administered at maximum rate of 1 g over an hour, which greatly decreases the likelihood of RMS occurring.

Skin prick testing (SPT) to vancomycin was not performed as vancomycin can directly release histamine from cutaneous mast cells [10], causing a false positive test and poor specificity. Successful SPT has been reported [11,12] but there is no validated protocol yet. Oral challenge consists of giving graded doses of the drug and has an inherent risk of anaphylaxis. We proceeded directly to oral desensitisation due to the likelihood of a reaction on challenge, which, given this patient’s comorbidities, would carry significant risk.

Vancomycin desensitisation is indicated in anaphylaxis and severe RMS when the drug is necessary. There are several published protocols for intravenous vancomycin desensitisation, but these are associated with a high incidence of significant reactions [7]. In general, oral desensitisation is considered to be safer than that via the intravenous route. The desensitisation protocol used in this case was developed with reference to a previously published protocol for intravenous vancomycin desensitisation. It was successfully administered in a controlled environment without adverse reactions.

## Conclusion

We suggest oral desensitisation to vancomycin to facilitate administration of this drug when required in a vancomycin allergic patient with severe or recurrent CDI, and outline a potentially safe and effective approach.

## Patient consent

Informed consent for treatment was obtained from the patient. The patient sadly passed away before submission of this case report and so consent to publish this case report could not be obtained. Having worked closely with the patient for many years, the doctors involved believe that the patient would not have had any objection to the case report being published. The Editor-in-Chief is satisfied that the conditions required to publish without consent have been met.

## Competing interests

The authors declare that they have no competing interests.

## Authors’ contributions

SM and RL carried out the procedure and drafted the manuscript. FF, CM and MK advised on patient care, and reviewed the manuscript. MK developed the protocol. All authors read and approved the final manuscript.
